# An integrated individual-level trait-based phytoplankton dataset from transitional waters

**DOI:** 10.1038/s41597-023-02785-w

**Published:** 2023-12-13

**Authors:** Maira Laraib, Jessica Titocci, Ilaria Rosati, Alberto Basset

**Affiliations:** 1 University of Salento, Department of Biological and Environmental Sciences and Technologies (DiSTeBA), Lecce, Italy; 2 Italian National Research Council (CNR), Institute for Research on Terrestrial Ecosystems (IRET), Lecce, Italy; 3 National Biodiversity Future Center, Palermo, 90133 Italy

**Keywords:** Limnology, Ecosystem ecology

## Abstract

Functional trait-based approaches have undergone an extraordinary expansion in phytoplankton ecology. Morpho-functional traits have been shown to vary both within and between populations and species, potentially affecting individual fitness and the network of inter-individual relationships. Here we integrate six fully harmonized phytoplankton morpho-functional trait datasets, characterized by a fine data grain, reporting individual-level data over a large biogeographical area. Datasets refer to transitional water ecosystems, from five biogeographical areas: Northern Atlantic Ocean (Scotland), South-Western Atlantic Ocean (Brazil), South-Western Pacific Ocean (Australia), Indo Pacific Ocean (Maldives) and Mediterranean Sea (Greece and Turkey). The integrated dataset includes 127311 individual phytoplankton records with sampling locations, taxonomic and morphometric information according to Darwin Core standards and semantic annotations. The six FAIR datasets are openly available in the LifeWatch Italy data portal. The datasets have already been used for morpho-functional analyses and hypothesis testing on phytoplankton guilds at different levels of data aggregation and scale, from local to global.

## Background & Summary

Trait-based approaches have become increasingly popular in community ecology^1,2^, including phytoplankton communities^3–6^, over the last few decades. Phytoplankton are a diverse group of microscopic organisms, accounting for approx. 40% of global primary productivity and are key contributors to the biogeochemical processes^7,8^. They provide an ideal model system for testing trait-based approaches, due to their relative simplicity and well-defined traits^9,10^. Phytoplankton morpho-functional traits affect the fitness and competitive success of individual cells, with cascading implications at the population, species and community levels^11^. Individual trait-based approaches provide the framework for linking individual responses to natural and anthropogenic pressures to community organization and ecosystem functioning^12,13^. Individual trait-based approaches have been applied in plant communities^14,15^ and more recently to plankton ones^16–18^. Here, we present an integrated individual-level trait-based phytoplankton dataset that combines six fully harmonized datasets related to transitional water ecosystems from the Northern Atlantic, South-Western Atlantic, South-Western Pacific, Indo Pacific Ocean and the Mediterranean Sea, sampled within the “Phytoplankton Bio-Imaging” project. All data are from a specific transitional water type, i.e., lagoon ecosystems, characterized by being micro-tidal, shallow and nutrient-rich depositional ecosystems^19^, determining morphometric traits adaptation of phytoplankton guilds when compared to the marine ones^20^. The lagoon ecosystems in each biogeographic area have been selected as relatively pristine ecosystems with low anthropogenic pressure; therefore, the integrated dataset as well as each individual biogeographic dataset can provide control/reference data for studying phytoplankton morphometric trait responses to anthropogenic pressure. The datasets have been harmonized and are openly accessible through the data portal of the Italian National distributed node (LifeWatch Italy) of the European e-Science Research Infrastructure LifeWatch ERIC. The integrated dataset presented here contributes to enhance the findable, accessible, and interoperable information^21^ on transitional water phytoplankton morpho-functional traits and complements the existing data resources on marine^22,23^ and freshwater^24–26^ phytoplankton.

## Methods

### Sampling and data collection

**Fig. 1:**
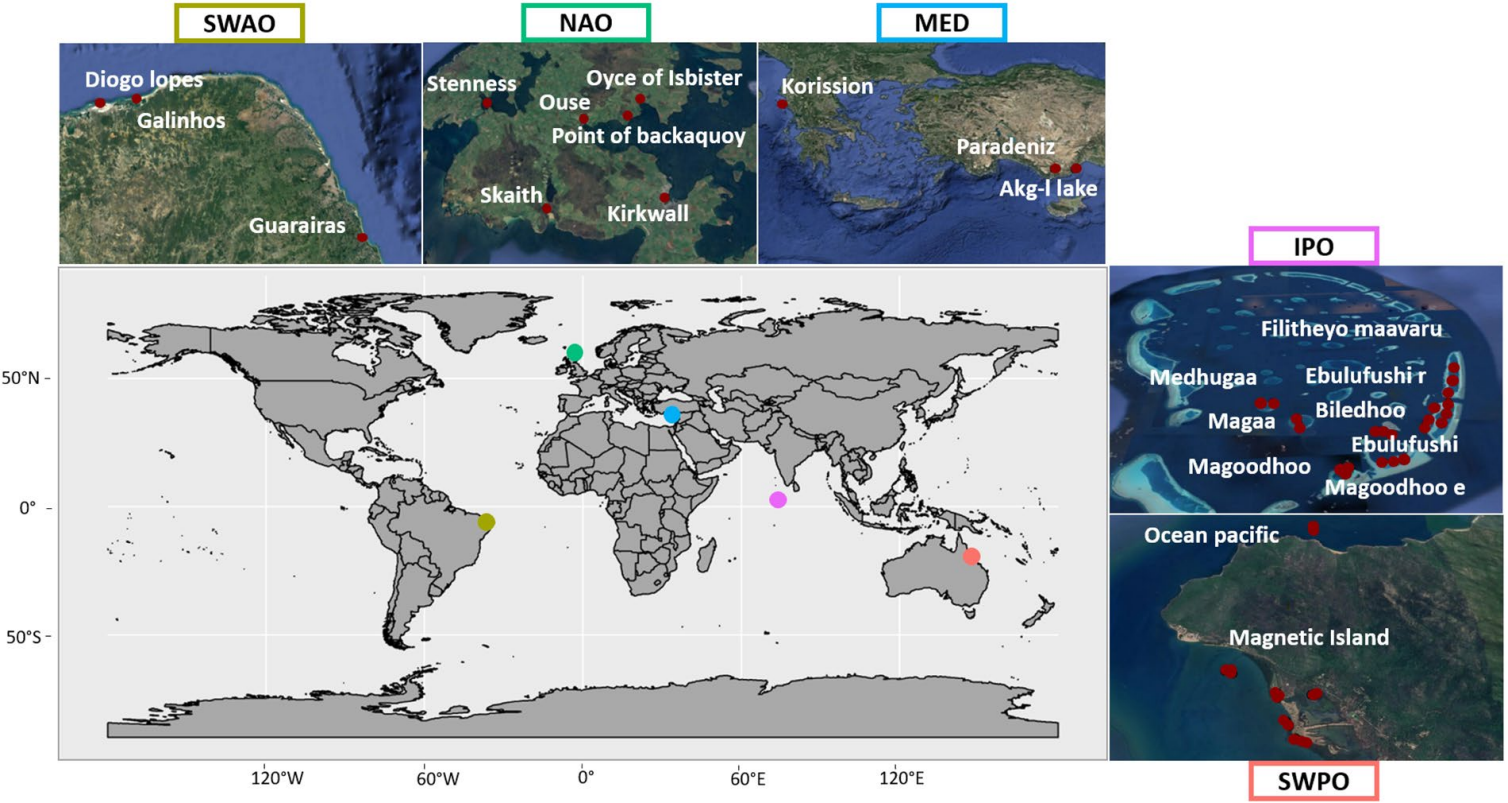
Distribution map of the five biogeographical areas included in the dataset: South-Western Atlantic Ocean (SWAO) in brown, Northern Atlantic Ocean (NAO) in green, Mediterranean Sea (MED) in light blue, Indo Pacific Ocean (IPO) in purple and South-Western Pacific Ocean (SWPO) in pink. The red dots identify the phytoplankton sampling stations in each biogeographical area.

Phytoplankton samples were collected in a single sampling event that took place between July 2010 and November 2012 in 24 transitional water ecosystems distributed across five biogeographical regions: Northern Atlantic Ocean (NAO-Scotland), South-Western Atlantic Ocean (SWAO-Brazil), South-Western Pacific Ocean (SWPO-Australia), Indo Pacific Ocean (IPO-Maldives) and Mediterranean Sea (MED- Greece and Turkey) (Fig. 1). Sampling was carried out according to a hierarchical sampling design: for each ecoregion, three ecosystems were selected and within each of these, a maximum of three habitat categories were chosen and three experimental stations per habitat type were sampled with three replicates each, for a total count of 116 sites and approximately 350 water samples. Habitat types were classified on the basis of sediment granulometry and type of bottom vegetation^27^ according to the EUNIS habitat type hierarchical classification, version 2012^28^.

Phytoplankton samples were collected with horizontal tows from the subsurface (0.5 m) using a net mesh (6 µm) and fixed with Lugol’s solution (15 mL/L). This sampling technique is not a 100% quantitatively; however, the sampling procedures were standardised following same protocol in every sampling campaign. During the net sampling phase, the net was towed from the boat for a standard length of approximately 1.5–2 m, repeated three times back and forth, with each haul consisting of a linear measure of approximately 10 m. Phytoplankton taxonomic identification, cell abundances estimations and morphometric measurements were performed using an inverted microscope (Nikon T300E, Nikon Eclipse Ti) connected to a video-interactive image analysis system (L.U.C.I.A Version 4.8, Laboratory Imaging), following the Utermöhl method^29^ at 400x magnification. For each sample, a minimum of 400 cells were counted, measured and identified to the lowest taxonomic level possible, using specific manuals, monographs and phytoplankton Atlas^30–37^. The taxonomic validation was performed using the World Register of Marine Species (WoRMS)^38^ and Algae Base^39^.Where identification to species level was not possible the “Cf.” qualifier was used to indicate a specimen relevant to the species claimed and the numbered “sp.” was used to denote an organism relevant to the identified genus.

After taxonomic identification, cell volumes (expressed in μm^3^) were estimated according to the species/taxa specific shape association and using the geometric equations for simple and complex shapes recorded in the webservice “Atlas of Shapes” https://www.phytovre.lifewatchitaly.eu/vre/shapes-groups/. The geometric shape was attributed to the shape of the individual cell, even for coenobial, colonial and filamentous species where cells were not observable. The cell and shape views (e.g., lateral, frontal, etc.) with all the corresponding measured linear dimensions were reported in the datasets using alphabetical codes (e.g. length indicated by “a”, “l”, etc.; width indicated by alphabetical code “b”, “d”, etc.), together with information on the presence of internal and external cell structures (Table 1). Cell volumes were also reported in the datasets as “volume equivalent to sphere” and “volume equivalent to cylinder” and calculated using the Nikon image analysis system, based on cell contours and the application of a rod model using minimum and maximum Feret distances as linear dimensions^40^. Phytoplankton cellular carbon content (pg C) was obtained indirectly by converting cell biovolume to carbon using empirical or theoretically derived equations in accordance with Menden-Deuer and Lessard, 2000^41^.

**Table 1:** Description of the dataset, according the Phytoplankton Data Template.

Column name	Definitions
catalogNumber	A unique identifier for each record within the dataset.
organismQuantity	An enumeration value for the quantity of organisms.
organismQuantityType	A quantification system used for the quantity of organisms.
eventID	A unique identifier for the associated information of an event (something that occurs at a place and time).
parentEventID	An identifier for the broader event information.
year	The four-digit year in which the event occurred.
month	The integer month in which the event occurred.
day	The integer day of the month in which the event occurred.
country	The name of the country in which the sampling location occurs.
countryCode	The standard code for the country in which the sampling location occurs.
locality	The specific description of the location.
decimalLatitude	The geographic latitude of the location lies between −90 and 90, inclusive.
decimalLongitude	The geographic longitude of the location lies between −180 and 180, inclusive.
phylum	The name of the phylum in which the taxon is classified.
class	The name of the class in which the taxon is classified.
family	The name of the family in which the taxon is classified.
order	The name of the order in which the taxon is classified.
genus	The name of the genus in which the taxon is classified.
providedScientificName	The scientific name with different qualifiers.
scientificName	The full scientific name identified in lowest level taxonomic rank.
measurementRemarks	Comments accompanying the measurement or fact.
Internal and External Structures	The measurable phenotypic characteristic of a cell or a colony related to physiology and ecology of organisms.
Shape	The approximate 3 dimensional geometric shape of the organism.
Biovolume	The volume of a single cell of each organism calculated according to the geometric equations associated with the measurements of linear dimensions (e.g., length, width, height) (Cubic micrometre per individual).
Cell Carbon Content	Cellular carbon content of each organism determined directly as particulate organic carbon or obtained indirectly by converting cell bio volume into carbon using empirically or theoretically derived equations (Picogram carbon per individual).
Linear dimensions (a,b,c,d,h)	a- The distance between two points indicated by alphabetical code “a, e, l”. In complex shapes, there are more than one length identified through different alphanumeric codes “a1, a2, a3, l1, l2, l3, l4”.
	b- Width is equal to the distance from one side of an object to another side “b, d”. In complex shapes, there are more than one width identified through different alphanumeric codes “b1, b2, b3, b4, d1, d11, d2, d22, d3, d33, d4, d44”.
	c- Thickness is equal to the dimension through an object as opposed to its length or width “c, h”. In complex shapes, there are more than one thickness identified through different alphanumeric codes “c1, c2, c3, h1, h2, h3, h4”.
VolumeeqSphere	Volume equivalent to sphere (cubic micrometers).
VolumeeqCylinder	Volume equivalent to cylinder (cubic micrometers).
ClassCode	In house numerical code linking taxonomic information to morphological computational volume and area calculation.
VolumeofSedimentation Chamber	The volume of water sample used for sedimentation expressed in milliliters.
TransectCounting	The number of count fields or diametric transect of the sedimentation chamber.
EunisHabitatsTypeName	Assignment of the habitat type name based on the EUNIS habitat classification.

The dataset attributes were labelled using terminologies from Darwin Core Standards and the Phytoplankton Trait Thesaurus.

## Data Records

The integrated dataset generated and analyzed for this study includes six datasets^42–47^ published in the LifeWatch Italy data portal https://dataportal.lifewatchitaly.eu/data with their respective DOIs (Table 2). The data were collected and harmonized according to the Phytoplankton Data Template https://www.phytovre.lifewatchitaly.eu/phyto-data-template/ which is based on the Darwin Core standards^48^ and the Phytoplankton Traits Thesaurus^49^. The datasets are formatted as column-oriented tables with data reported in semicolon_separated values format (.csv). The associated metadata are described using the Ecological Metadata Language^50^ (EML 2.2.0) standard in extensible markup language (.xml) format to ensure data understanding and long-term control. Each phytoplankton record is represented by an identifier (catalogNumber) associated with ancillary information (e.g. sampling locations, temporal and spatial information), phytoplankton taxonomic classification and morphological trait data (Table 1). Data variables are numeric and categorical and are expressed in text and numeric formats.

**Table 2:** List of datasets and respective DOIs.

Biogeographical areas	Dataset names	DOIs
South-Western Atlantic Ocean (SWAO)	Phytoplankton__Progetto_Strategico_2009_2012_Brasil42	10.48372/dc6c5838-0e81-4aac-9442-fe9cb0bdb604
Northern Atlantic Ocean (NAO)	Phytoplankton__Progetto_Strategico_2009_2012_United_Kingdom43	10.48372/5901dc22-9943-4fe0-9c23-1aeba0d52293
Mediterranean Sea (MED)	Phytoplankton__Progetto_Strategico_2009_2012_Greece44	10.48372/098f6be3-8d79-4797-b0d6-5b22cdec9829
	Phytoplankton__Progetto_Strategico_2009_2012_Turkey45	10.48372/4cf276c3-ba35-44f5-8ef0-a79de3e3bc06
Indo Pacific Ocean (IPO)	Phytoplankton__Progetto_Strategico_2009_2012_Maldives46	10.48372/e7e415b4-4d4f-4180-8880-0f9446970f39
South-Western Pacific Ocean (SWPO)	Phytoplankton__Progetto_Strategico_2009_2012_Australia47	10.48372/4ea04557-8431-4b2e-8dff-c15a11fa937a

**Fig. 2:**
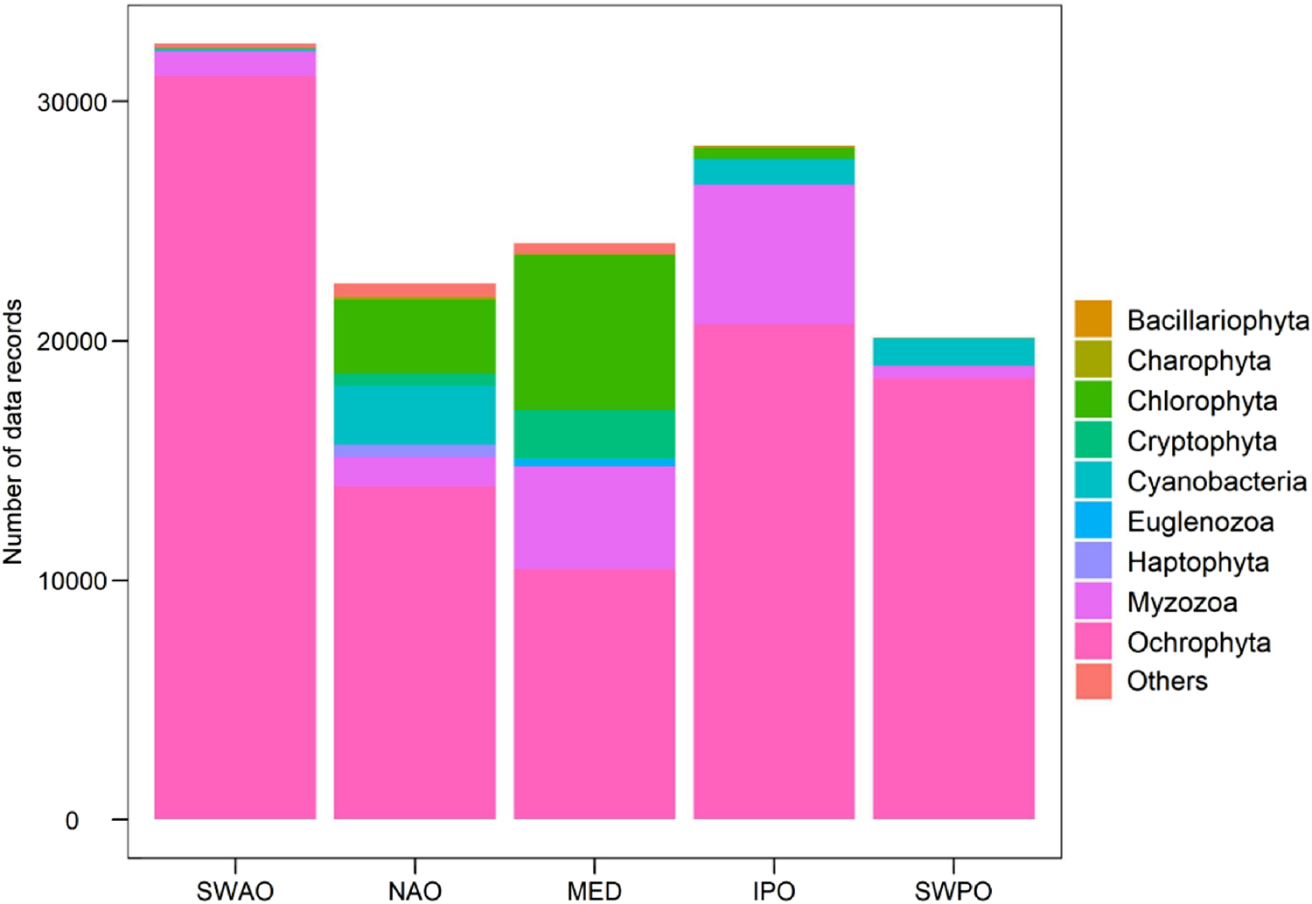
Barplot showing the distribution of phytoplankton data records per phyla in each biogeographical area: South-Western Atlantic Ocean (SWAO) Northern Atlantic Ocean (NAO), Mediterranean Sea (MED), Indo Pacific Ocean (IPO) and South-Western Pacific Ocean (SWPO).

**Fig. 3:**
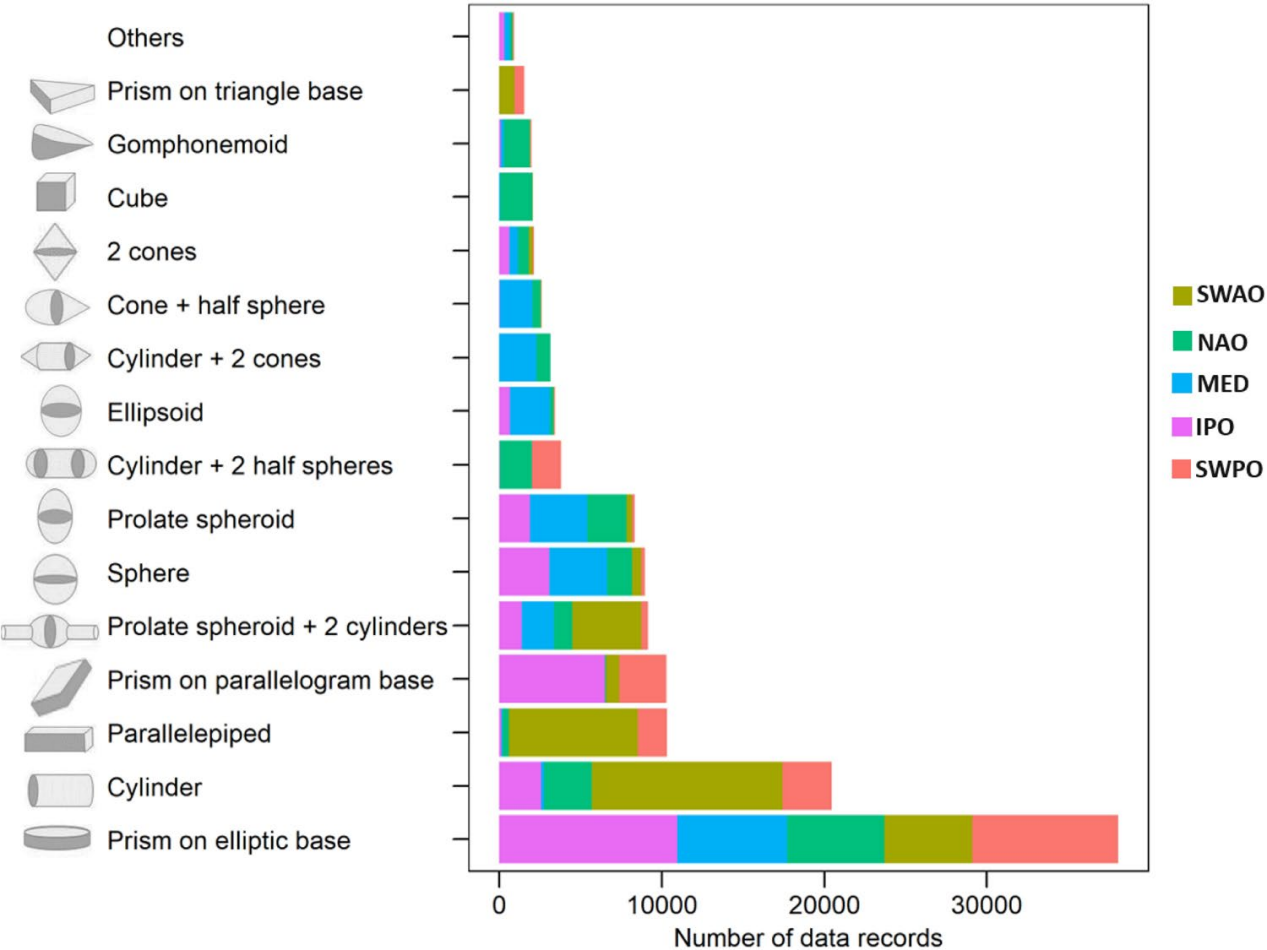
Barplot showing the number of phytoplankton data records and the number of shapes per biogeographical area: South-Western Atlantic Ocean (SWAO) in brown, Northern Atlantic Ocean (NAO) in green, Mediterranean Sea (MED) in light blue, Indo Pacific Ocean (IPO) in purple and South-Western Pacific Ocean (SWPO) in pink. The shape category “Others” refers to less representative complex shapes described according to the Atlas of Shapes: half ellipsoid + cone on elliptic base, prism on triangular base 1, cone, prism on elliptic base + 4 cones, ellipsoid + cone, 2 half ellipsoids, prism on elliptic base + parallelepiped, 2 half ellipsoids + prism on elliptic base, ellipsoid + 2 cones + cylinder, cymbelloid, pyramid on rectangular base, cylinder + 3 cones, half sphere, parallelepiped + 2 cylinders, half ellipsoid + 3 cones, parallelepiped + 6 half cylinders, truncated cone, truncated cone + truncated cone, prism on elliptic base + 2 parallelepipeds and sickle-shaped prism.

In total 127311 phytoplankton cells, belonging to 306 taxa were counted, measured and taxonomically classified. Summarized information from each dataset is presented in Table 3. The highest abundance was recorded in South-Western Atlantic Ocean area (SWAO), while the lowest number of records was reported in the South-Western Pacific Ocean (SWPO) region. In terms of taxa richness and shape occurrence a rather similar trend occurred in all biogeographical areas, with Northern Atlantic Ocean (NAO) biogeographical area showing the highest diversity in terms of taxa composition and the Indo Pacific Ocean area (IPO) showing the highest diversity in terms of shape occurrence. The distribution of phytoplankton composition by phyla in each biogeographical area showed a noticeable predominance of Ochrophyta in all regions (Fig. 2) mainly represented by the genera *Chaetoceros*, *Pseudo-nitzschia*, *Ceratoneis*, *Cyclotella*, *Thalassionema* and *Navicula*, followed by the Myzozoa represented by 66 different taxa. Other phyla such us Chlorophyta, Cyanobacteria, Cryptophyta, Haptophyta, Euglenozoa, Charophyta, Bacillariophyta and others accounted for less than 10000 individuals in each phyla, for a total of 87 taxa. A total count of 35 different morphological shapes of phytoplankton were described in the integrated dataset. Prism on elliptic base was the most abundant shape in terms of data records in all the biogeographical areas examined (38073 records) except for the South-Western Atlantic Ocean (SWAO) region where cylinder, parallelepiped and prolate spheroid + 2 cylinders were the most dominant shapes (Fig. 3). Complex shapes such as cylinder + 2 cones and cone + half sphere were mainly found in Northern Atlantic Ocean (NAO) and Mediterranean Sea (MED) regions with more than 2000 phytoplankton individuals, while more than 1500 organisms with cubic and gomphonemoid shapes were recorded in Northern Atlantic Ocean (NAO) area. The category “others” which included a mix of 20 different simple and complex shapes, rarely contributed to the overall morphological distribution of phytoplankton, with less than 1000 individuals.

**Table 3:** Summary of total abundance, taxa richness and shape occurrence from five biogeographical areas.

Biogeographical areas	Abundance (n. of cells)	Taxa Richness	Shape occurrence
South-Western Atlantic Ocean (SWAO)	32400	96	21
Northern Atlantic Ocean (NAO)	22396	120	20
Mediterranean Sea (MED)	24080	110	19
Indo Pacific Ocean (IPO)	28296	116	23
South-Western Pacific Ocean (SWPO)	20139	114	19

## Technical Validation

**Fig. 4:**
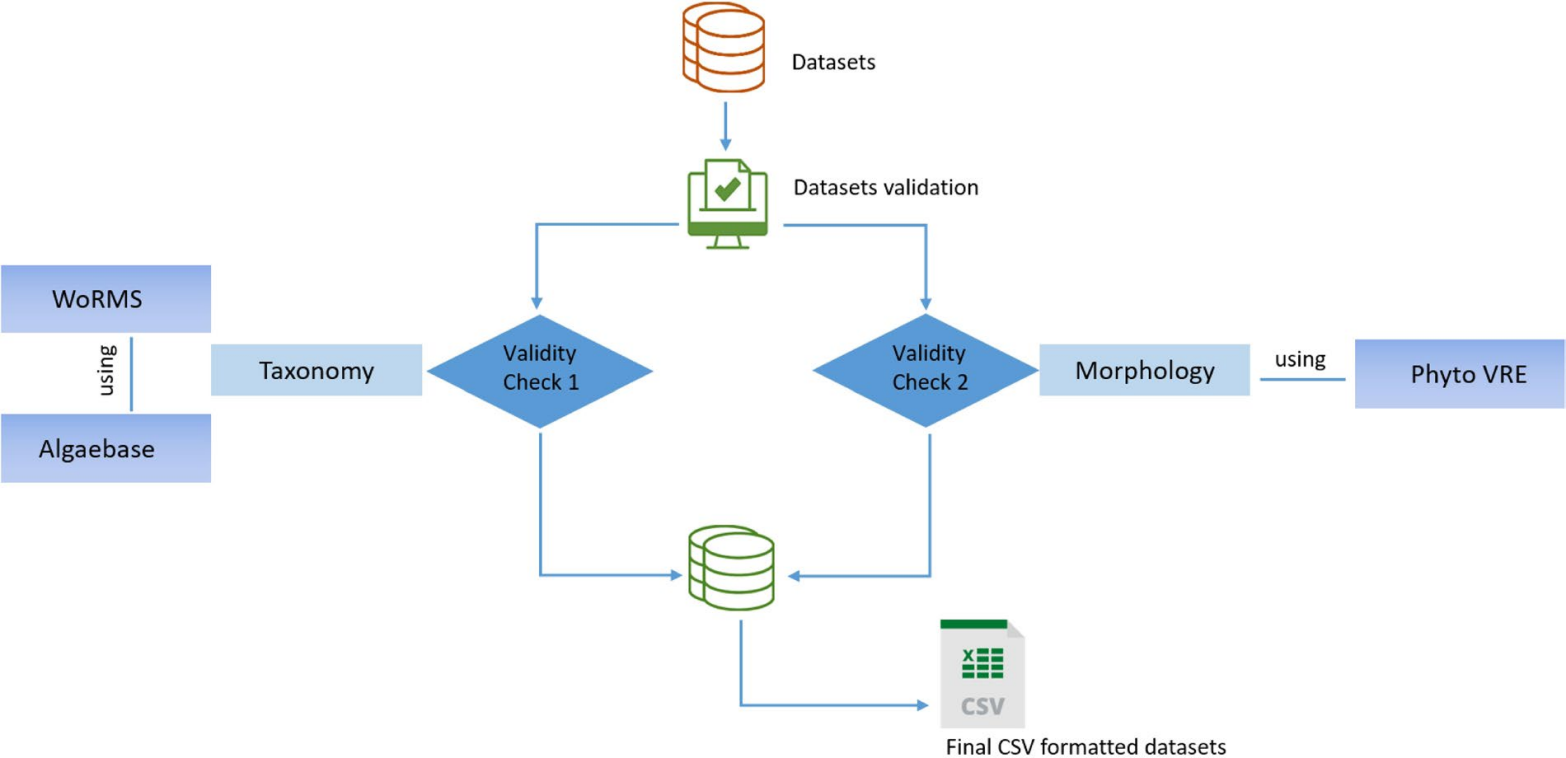
Schematic illustration of the data validation process. Taxonomical and morphological information were checked and technically validated through the use of WoRMS and Algaebase repository and the web services of the Virtual Research Environment “PhytoVRE”.

Data curation and technical validation steps were carried out to ensure the accuracy of the data and metadata. During data collection, a standardized sampling protocol already used in previous studies^51^ and the same sampling design were followed throughout the entire sampling campaign to avoid bias and ensure replicability of the data and information. Secondly, all the samples were collected, identified and measured by a team of qualified researchers and taxonomists who ensured data quality by checking and validating the taxonomic and morphological classification of the phytoplankton and detecting format and nomenclature errors or missing and inconsistent data. Thirdly, taxonomical and morphological information contained in all datasets were checked and technically validated through the use of WoRMS and Algaebase repository and the web services “Atlas of Shapes” and “Trait computation” provided in the Virtual Research Environment “Phyto VRE” of LifeWatch Italy (Fig. 4). After all the curation and validation steps, the data were stored and preserved in the LifeWatch Italy data portal, making them findable, accessible, interoperable and reusable. Finally, the integrated dataset includes information that have already been published in peer-reviewed scientific journals^52–58^.

## Data Availability

The provided datasets were established without the use of any custom code.
